# Intention to Take COVID-19 Vaccine as a Precondition for International Travel: Application of Extended Norm-Activation Model

**DOI:** 10.3390/ijerph18063104

**Published:** 2021-03-17

**Authors:** Aleksandar Radic, Bonhak Koo, Eloy Gil-Cordero, Juan Pedro Cabrera-Sánchez, Heesup Han

**Affiliations:** 1 Independent Researcher, Gornji kono 8, 20 000 Dubrovnik, Croatia; aleradic@gmail.com; 2 School of Hospitality and Tourism Management, Spears School of Business, Oklahoma State University 365 Human Sciences, Stillwater, OK 74078, USA; bkoo@okstate.edu; 3 Department of Business Administration and Marketing, Universidad de Sevilla, 41018 Sevilla, Spain; egcordero@us.es (E.G.-C.); jcabrera10@us.es (J.P.C.-S.); 4 College of Hospitality and Tourism Management, Sejong University, 98 Gunja-Dong, Gwanjin-Gu, Seoul 143-747, Korea

**Keywords:** COVID-19 mandatory vaccination, Norm-Activation Model, mass media coverage, behavioral intention

## Abstract

The COVID-19 pandemic is a serious threat to human health, the global economy, and the social fabrics of contemporary societies as many aspects of modern everyday life, including travel and leisure, have been shattered to pieces. Hence, a COVID-19 mandatory vaccination as a precondition for international travel is being debated in many countries. Thus, the present research aimed to study the intention to take the COVID-19 vaccine as a precondition for international travel using an extended Norm-Activation Model. The study model integrates a new construct, namely mass media coverage on COVID-19 vaccination as additional predictor of intention to take the COVID-19 vaccine. The survey data were collected from 1221 international travelers. Structural equation modelling shows a very good fit of the final model to the data; the conceptual model based on extended Norm-Activation Model was strongly supported. Awareness of consequences related to the COVID-19 pandemic on individuals’ health has shown a positive effect on individuals’ ascribed responsibility to adopt emotionally driven (anticipated pride and anticipated guilt) pro-social behaviors that activate a personal norm towards altruistic and pro-mandatory vaccination-friendly behavior. Theoretical and practical implications are discussed.

## 1. Introduction

The COVID-19 pandemic is a serious threat to human health, the global economy, and the social fabrics of contemporary societies as many aspects of modern everyday life, including travel and leisure, have been shattered to pieces. In 2020, global tourism experienced devastation of epic proportions as international arrivals dropped 74% compared to 2019, leading to a loss of USD 1.3 trillion in export revenues [1]. Governments across the globe are engaging in national lockdowns and travel restrictions as tactics of a comprehensive strategy to counteract the COVID-19 pandemic. However, such actions are becoming unbearable for many people. As Wilson and Chen [2] argued, travel is a constitutive element of modern human lives. Hence, many countries are debating a COVID-19 mandatory vaccination as a precondition for international travel. Accordingly, a COVID-19 mandatory vaccination as a precondition for international travel might become a new social dividing line. International travelers who have been COVID-19-vaccinated will likely encounter fewer travel barriers, while those who have not been COVID-19-vaccinated might remain subject to travel restrictions, quarantining, and testing. Furthermore, Laurent [3] argues how the travel industry drives such an idea as a leap towards normality after the collapse of international travel in 2020 and technology enterprises long for profitable government contracts and a golden goose of personal data.

COVID-19 vaccination as a precondition to international travel was suggested by Gössling et al. [4], who argued that the cruise industry is doomed without an efficient COVID-19 vaccine. Accordingly, as major cruise lines plan to make the COVID-19 vaccination mandatory for their crew members, their legal teams explore the possibilities of enforcing such requirements for guests [5]. Saga Cruises have gone a step further and recently announced that they will require all guests to be fully vaccinated [6]. Furthermore, in a recent survey by Cruise Critic, 81% out of 3000 participants (cruisers) said they would cruise if COVID-19 vaccines were required [7]. Looking at 2021 global international travel, Read [8] outlined that COVID-19 vaccination might not be mandatory. However, it will certainly create favorable conditions for international travel for those who are vaccinated. Qantas was the first airline to announce that it will require international passengers to be vaccinated. Moreover, Phelan [9] outlined that COVID-19 vaccination, followed by immunity passports as a precondition for international travel, may cause discriminatory consequences if the World Health Organization does not revise its recommendations for the COVID-19 Public Health Emergency of International Concern in the International Health Regulations Annex 7. Accordingly, Hatz et al. [10] concluded that the COVID-19 pandemic clearly demonstrates the importance of vaccinations for international travel, and potential travelers can expect to obtain country-specific requirements in the near future such as “immunity passports” and proof of vaccination. Lastly, in a recent interview with the World Travel and Tourism Council, CEO Gloria Guevara categorically stated that she is against COVID-19 vaccination as a precondition for international travel as such action could create discrimination [11].

The pioneering study described here sets out to investigate the intention to take the COVID-19 vaccine as a precondition for international travel. More precisely, this research sets to extend the Norm-Activation Model (NAM) [12] by exploring the relationships between mass media coverage and awareness of consequences and behavioral intention.

To accomplish this task, the following research question is addressed: “Will travelers take the COVID-19 vaccine as a precondition for international travel?”

Intending to enhance academic literature on the topic of behavioral intentions towards traveling in the time of the COVID-19 pandemic, the authors proposed three main research objectives. In particular, the present research aimed (1) to test if the extended Norm-Activation Model (NAM) could explain altruistic and mandatory vaccination-friendly behavior, (2) to explore the effect of mass media coverage on awareness of consequences and behavioral intention, (3) to identify the respective significance between constructs within the proposed theoretical framework in determining pro-mandatory vaccination intentions. The present research has a theoretical value and originality as, to the authors’ best knowledge, it is the first study that has applied the NAM to the context of mandatory vaccination as a precondition for international travel. Practically, this study can assist policymakers and governments in understanding the sentiment behind potential mandatory COVID-19 vaccination as a precondition for international travel.

## 2. Literature Review

### 2.1. Norm-Activation Model (NAM)

The essence of the Schwartz’s NAM [12] lies in the function of personal morals, as personal beliefs are crucial to one’s behavior. More precisely, Schwartz proposed that awareness of consequences and ascription of responsibilities are conditions for the activation of personal norms [12]. Awareness of consequences relates to the individuals’ mindfulness about detrimental reaction/consequences when not performing pro-socially for others or in addition to one’s values. Hence, the individuals’ understanding of consequences for not acting pro-socially has to be robust and meaningful so the person can acknowledge that others need help and that their ability to engage pro-socially could help others. Ascription of responsibility relates to contemplation on sentiments concerned with responsibility for the adverse consequences of not engaging pro-socially [13]. Thus, individuals need to take on certain responsibility for the issue that is presented so they could become engaged and provide assistance. Personal norm indicates an obligation arising out of considerations of engaging or withholding from certain activities [14]. Personal norms are closely bound with one’s self-concept; hence, breaching them brings guilt, and bracing them grows pride [15]. Pride and guilt are termed as self-conscious emotions since they incorporate self-evaluation [16]. Accordingly, pride and guilt are evoked by the awareness that particular actions have led to the violation or tension in an important relationship or community [17]. Moreover, once social norms are put into effect at the personal level, they become personal norms. The NAM’s peculiarity is that it takes to scrutiny that even though people verbally agree with a norm that has authority on certain moral behavior, not all of them act suitably. The NAM is a psychological behavioral model that has been used to a large degree in the context of travel behavior [18,19,20,21]. In the context of convention attendance, Han [18] concluded that anticipated feelings of pride or guilt positively affected convention travelers’ personal norms. Ascribed responsibility, anticipated emotions, and the personal norm had a significant mediating impact on attending environmentally responsible conventions. Furthermore, in the smart tourism context, Kiatkawsin et al. [19] have confirmed that the involvement of culture and attitude towards cultural conservation positively affects tourists’ environmentally responsible behavior. In their study on drone food delivery services, Kim and Hwang [20] outlined the importance of the moderating role that product knowledge played in eco-friendly behavioral intention. In a recent meta-analysis by Manosuthi et al. [21], authors outlined that subjective or social norms are triggered by the personal norm, while the personal norm has a mediating role during the formation of revisit intention of the young tourists engaged in pro-social or pro-environment tourism.

In the field of tourism and hospitality, awareness of consequences, ascribed responsibility, anticipated pride, anticipated guilt, and personal norms have been empirically proven to be essential in understanding tourist pro-social behaviors [18,19,20,22,23,24,25,26]. The positive effect of awareness of consequences on ascribed responsibility was confirmed with young travelers’ intentions to behave pro-environmentally [22], pro-environmental behavior of tourists engaged in green lodging [23], and smart tourists’ environmentally responsible behavior [19]. Kim and Hwang [20], in their recent study on drone food delivery services, confirmed that ascribed responsibility positively influenced personal norms. The ascribed responsibility significantly predicted pro-environmental personal norms of tourists visiting national parks [24]. Moreover, Liu et al. [25] argued that the competitiveness of tourism destinations lies in the tourists who behave in a civilized manner, where personal norm plays a significant role in driving civilized behavioral intentions. Individuals who engage in last-chance tourism are driven by their personal norms, which also influence their intention to behave in pro-sustainable ways [26]. In Han’s [18] study on decision-making in environmentally responsible convention attendance, the author concluded that ascribed responsibility has a positive effect and influence on anticipated feelings of pride; however, it has the negative effect of the anticipated feeling of guilt. The aforementioned author in the same study confirmed that an anticipated feeling of pride positively affects personal norm, while an anticipated feeling of guilt negatively affects personal norm [18].

Previous studies have provided valuable theoretical and managerial contributions. However, the COVID-19 pandemic has changed the international travel landscape; hence, this study possesses originality as it offers important novel theoretical contributions. Based on a review of the academic literature and relevant empirical studies, the authors proposed the following hypotheses:

**Hypothesis** **1** **(H1).** *Awareness of consequences has a positive impact on ascribed responsibility*.

**Hypothesis** **2** **(H2).** *Ascribed responsibility has a positive impact on personal norms*.

**Hypothesis** **3** **(H3).** *Personal norms have a positive impact on intention to take the COVID-19 vaccine before international travel*.

**Hypothesis** **4** **(H4).** *Ascribed responsibility has a positive impact on anticipated pride*.

**Hypothesis** **5** **(H5).** *Anticipated pride has a positive impact on personal norm*.

**Hypothesis** **6** **(H6).** *Ascribed responsibility has a negative impact on anticipated guilt*.

**Hypothesis** **7** **(H7).** *Anticipated guilt has a negative impact on personal norm*.

### 2.2. Mass Media

Mass media is a complex organization that uses standardized practices to produce public messages distributed in a relatively short time, or even simultaneously, to a widely dispersed population [27]. During crisis and normal times, mass media has an instrumental function as it provides essential, applicable information that swiftly guides the audience’s engagement [28]. The effects of mass media have longstanding consequences on individuals’ behavior, worldviews and values, as well as learning abilities [29]. Mass media is used both in travel and tourism and in public health in order to influence peoples’ health choices and shape their destination choices. Mass media possess the robust influence on individuals’ public health perception as impacts on population health can take a part through intentional efforts by public health agencies to disseminate potential risks, prevention and treatments [28]. Accordingly, mass media influence on public health perception is delivered through social context and macrosocial arch that influence the individuals’ behavior [28]. Furthermore, based on the Katz and Lazarsfeld [30] multi-step flow theory, it is evident that mass media coverage on COVID-19 vaccination and pro-social behavior in the form of taking the COVID-19 vaccine is driven by notable formal and informal high-level opinion leaders. The importance of mass media during the COVID-19 pandemic was confirmed by Harapan et al. [31], who outlined that acceptance of a COVID-19 vaccine in Southeast Asia is influenced by knowledge of the disease. Similarly, Leng et al. [32] argued how mass media can improve vaccination coverage through rapid and broad communication regarding local vaccine coverage. Mass media health campaigns can positively influence individuals’ attitudes and beliefs as they are often vividly recalled [33]. Thus, advocating the vaccination through the mass media is of paramount importance in any pro-vaccination strategy [34]. In travel and tourism, mass media possess a robust influence on the destination image and people’s awareness as it strongly affects their visit intentions [35]. Moreover, Kiatkawsin et al. [19] argued that mass media has strong influences on the behavioral characteristics of smart tourists, as smart tourists are highly engaged on social media platforms and are susceptible to the mass media influence. Volunteer tourists often share their attractive travel pictures that sketch the joy and happiness of the local community on mass media. Such actions possess the capacity to activate the moral norm of other potential tourists [21]. Mass media’s effect is evident in green international convention tourism as mass media holds a strong positive influence on participants’ attitude [36]. In a recent study by Radic et al. [37] on intention of female passengers to dine on board cruise ships in the time of the COVID-19 pandemic was negatively affected by the mass media coverage. Mass media coverage can affect the perception of audience awareness and behavior [38] and the full strength of mass media coverage influence on audience behavior is evident in the current COVID-19 pandemic [39,40,41].

From the aforementioned studies, it can be concluded that mass media’s influence on framing the intention to take the COVID-19 vaccine as a precondition to travel is non-existent to this date. Thus, this study is unique as it offers a new perspective for the uncharted academic space by offering valuable theoretical and practical contributions. Consequently, it can put forward the following hypotheses:

**Hypothesis** **8** **(H8).** *Mass media coverage has a positive impact on awareness of consequences*.

**Hypothesis** **9** **(H9).** *Mass media coverage has a positive impact on the intention to take a mandatory COVID-19 vaccine before international travel*.

**Figure 1:**
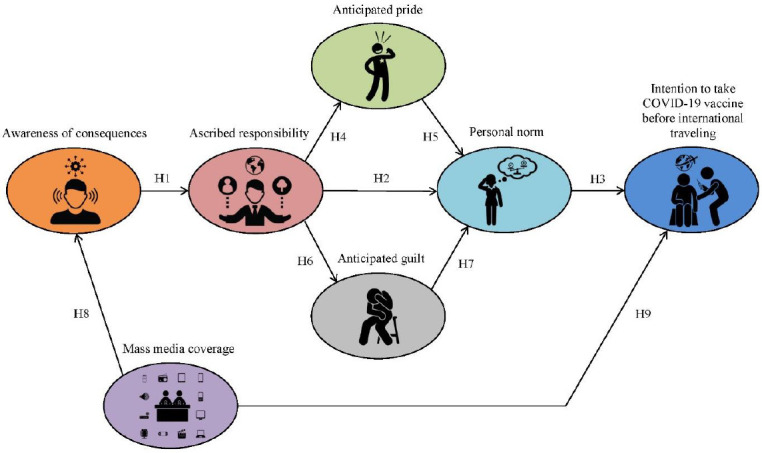
The conceptual model based on extended Norm-Activation Model (NAM).

In summary, the authors proposed a research model that integrates NAM fundamental variables of (awareness of consequences, ascribed responsibility, anticipated pride, anticipated guilt, personal norms, and behavioral intention) extended by the effect of mass media coverage on awareness of consequences and behavioral intention. A conceptual model of these relationships is presented in Figure 1.

## 3. Method

### 3.1. Measures

The survey contained a mix of multi-item and one dual-item measures (see Appendix A for a complete list of items). Measurement items for the constructs of the NAM, which include awareness of consequences, ascribed responsibility, anticipated pride, anticipated guilt, personal norms, and intention to take the COVID-19 vaccine before international travel, were adopted and adjusted from Han’s [18] scale. Measurement items for the constructs of the mass media coverage were adopted from the scale of Juschten et al. [35]. Academic experts reviewed and improved the initial version of the survey questionnaire, including these measurement items and questions about personal characteristics, accordingly. The common method bias of self-administrated questionnaires was minimized by following the methods of Podsakoff et al. [42]. Furthermore, the method of Terglav et al. [43] was used to avoid the replication of the hypothesis structure.

### 3.2. Sample and Data Collection Procedure

In order to assess tourist intention to take the COVID-19 vaccine as a precondition for international travel, data were collected via self-administrated online survey cloud-based software (SurveyMonkey^®^) during the period between December 2020 and January 2021. The potential weaknesses of online survey were eliminated and in some areas moderated by following Evans and Mathur’s [44] set of guidance. A purposive sampling technique was applied in this study as Teeroovengadum and Nunkoo [45] argued that purposive sampling techniques produce well-suited participants established on the authority of the chosen criteria. Possible participants were invited to take part in the survey via various social media groups (please see Table 1).

Potential participants were introduced to the purpose of the study via a short explanatory section that was incorporated into the survey. Individuals who understood the purpose of the study and agreed to participate in the survey qualified to continue with the screening questions of the questionnaire. Screening questions were used to verify that only those tourists who had traveled internationally before the COVID-19 pandemic participated in the survey. Participants were asked to answer on a nominal scale (“yes” or “no”) to the following question, “Have you traveled internationally in the last 24 months?” A total of 1403 questionnaires were initially collected, and the average time taken by respondents to complete each questionnaire was 8 min.

### 3.3. Data Screening

Data screening was conducted to ensure that the collected data were well prepared for further statistical analyses. First, univariate outliers were checked by standardized values (z-scores), and there were no absolute values that exceeded 3.29. Second, multivariate outliers were detected by Mahalanobis distance (MD), and the probability of MD below 0.001 was removed (MD (22) > 48.102, *p* < 0.001). Third, the common method bias (CMB) was checked by Harman’s single-factor analysis. The results showed that total variance did not exceed 50%. Finally, 1221 responses were utilized for data analysis.

## 4. Results

### 4.1. Results of the Demographic Information

The sample was composed of 1221 participants (see Table 2) from various regions across the globe (19% from Southeast Asia, 17.6% from China, 17.1% from Africa, 13.9% from South Asia, 11.2% from North America, 10.2% from Europe, 10% from Central/South America and 1% from Australia and New Zealand). The majority of participants were between 20 and 29 years old (39.7%), 30 and 40 years old (30.6%), and 40 and50 years old (22.7%). Regarding the education level, 40.3% indicated that they had a bachelor’s degree, 22.6% specified that they had a high school diploma, 20.6% indicated that they had an associate degree, while 16.7% had a master’s or Ph.D. degree. Lastly, when it comes to gender, 51.7% were female, and 48.3% were male.

### 4.2. Results of the Measurement Model

This study utilized structural equation modeling (SEM) to test the proposed theoretical model. SEM is a covariance-based structure analysis that allows researchers to test multivariate data analysis. SEM is regarded as the most appropriate statistical approach as it overcomes the shortcomings of traditional statistical methods by explicitly detecting measurement errors [46]. SEM consists of two distinct phases. The first phase is to test the measurement model, which examines the relationships between the latent variables and their measures. This test allows researchers to determine whether a priori theory fits the proposed theoretical model within the target population. The second phase aims to test the fully latent structural model, which is intended to identify the direct/indirect relationships between the latent variables. In other words, the fully latent structural model helps to test the proposed hypotheses.

The maximum likelihood (ML) was utilized as a method of estimation as the data are assumed to be normally distributed with sufficiently large samples. MPLUS v.8.4 [47] was utilized as a computer tool.

The results of the confirmatory factor analysis (CFA) shows that the global fit of the proposed model is satisfactorily presented (χ^2^ (131) = 625.377, *p* < 0.001, RMSEA = 0.056 [0.051, 0.060], CFI = 0.977, TLI = 0.970, SRMR = 0.019). Reliability and validity were checked next. First, internal consistency reliability was confirmed by Cronbach’s alpha and composite reliability (CR), which ranges from 0.846 to 0.945, and 0.848 to 0.945, respectively [48]. Second, the validity of the model was checked by average variance extracted (AVE). The results show that all values exceeded the recommended minimum value of 0.50 [49], ranging from 0.650 to 0.853. In this regard, the measurement model results imply that the data satisfactorily fit the proposed theoretical model and were ready to test the structural model (see Table 3 and Table 4 for more details).

### 4.3. Structural Model and Hypotheses Testing

The structural model was analyzed for hypotheses testing. The global fit of the structural model shows that the proposed model fit the collected data successfully (χ^2^ (141) = 888.142, *p* < 0.001, RMSEA = 0.066 [0.062, 0.070], CFI = 0.965, TLI = 0.958, SRMR = 0.084). The results of the hypotheses test indicated that all of the hypotheses were supported except for hypothesis 4. In detail, awareness of consequences positively influenced ascribed responsibility (β 0.833, *p* < 0.001); thus, hypothesis 1 was supported. Ascribed responsibility positively influenced personal norm (β 0.047, *p* < 0.01), while ascribed responsibility negatively influenced anticipated guilt (β −0.439, *p* < 0.001); thus, hypotheses 2 and 6 were supported. However, ascribed responsibility had no significant impact on anticipated pride (β −0.005, *p* > 0.05). In other words, hypothesis 4 was not supported. Findings also indicated that personal norm positively influenced behavioral intention (β 0.943, *p* < 0.001) and anticipated pride positively influenced personal norm (β 0.938, *p* < 0.001); thus, hypotheses 3 and 5 were supported. Lastly, mass media coverage positively influenced both awareness of consequences (β 0.194, *p* < 0.001) and behavioral intention (β 0.063, *p* < 0.001); thus, hypotheses 8 and 9 were supported.

**Figure 2:**
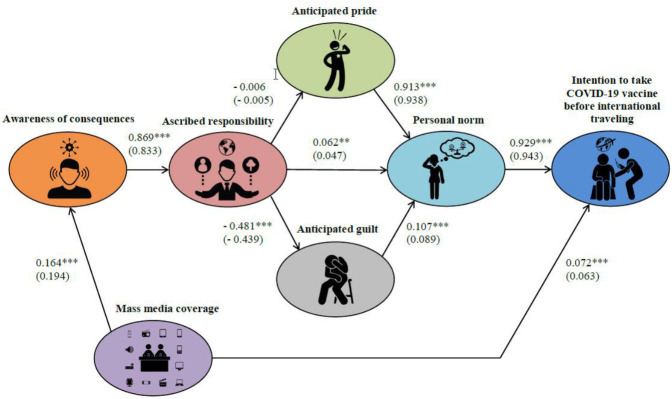
Results of the structural model estimation (n = 1221). Note. Goodness-of-fit indices: χ2 (141) = 888.142, *p* < 0.001, RMSEA = 0.066 [0.062, 0.070], CFI = 0.965, TLI = 0.958, SRMR = 0.084. ** *p* < 0.01, *** *p* < 0.001.

Next, the indirect effect of ascribed responsibility and personal norm in the relationship between awareness of consequences and behavioral intention was tested to provide a supplementary implication from the model. The bias-corrected bootstrapping technique was used to increase the accuracy of estimates. The results show that awareness of consequences indirectly affected behavioral intention through the intermediary variables of ascribed responsibility and personal norm (β 0.37, *p* < 0.01). Furthermore, the percentage of explained variance (*R*^2^) shows that the predictors explain about 89% of the variance in behavioral intention. Table 5 and Figure 2 show the results of the fully latent structural regression model.

## 5. Conclusions

The COVID-19 pandemic ravaged global international travel in 2020, and the rebound of international tourism in 2021 is still very much uncertain. Accordingly, the authors have assessed the likelihood of the NAM in clarifying the mandatory vaccination as a precondition of international traveling behavior in hospitality and tourism. This research aimed to test the robustness of the NAM and extend this model by incorporating certain constructs that are pivotal in mixed mandatory COVID-19 vaccination-friendly behavior in an international travel context. In summary, the conceptual model-based extended NAM was strongly supported. More precisely, the conceptual model demonstrated a very good fit to the data and the potency in predicting travelers’ intention to take a mandatory COVID-19 vaccine as a precondition to international travel. The hypotheses relationships between study constructs were, all but one, supported, and the noteworthy capacity of mass media coverage and personal norm in setting behavioral intention was evident.

In this study, ascribed responsibility did not have a significant positive impact on anticipated pride. This can be understood, as it appears that an individual’s freedom of choice is overlapping with potential government-enforced actions. Although an individual’s responsibility is based on his/her moral compass, stripping one’s sovereignty over himself/herself removes one’s feeling of pride. Hence, anticipated pride that could be induced by the solicitude of adverse consequences is most likely diminished due to the lack of freedom of choice. This finding is supported by Sartre [50], who argued that freedom penetrates each and every facet of the human condition, as the sole existence is freedom. Moreover, every single human being has freedom of choice. Thus, it is this choice that sets apart each individual’s *being* [50]. Consequently, as the feeling of pride from achievement and the feeling of guilt from failure are features of the self [51], this study demonstrated that even though individuals do not take pride from ascribed responsibilities, however, they feel guilt. Finally, as pride is a composite secondary emotion based on the expansion of a sense of self, Dawkins [52] outlined that human beings have to be taught altruism as every single one of us is born selfish.

Awareness of consequences related to the COVID-19 pandemic on an individual’s health has shown a positive effect on an individual’s ascribed responsibility to adopt emotionally driven (anticipated pride and anticipated guilt) pro-social behaviors that activate a personal norm towards altruistic and pro-mandatory vaccination-friendly behavior. More precisely, awareness of consequences is related to the individual’s aim, which is, in this case, ascribed responsibility towards avoiding the disease and protecting others from the disease. Furthermore, ascribed responsibility holds the potential to build courage in people, so they voluntarily take on the heavy load of being and acting in accordance with their personal norm. Personal norm colors an individual’s perception of self-consciousness and ultimately steers behavioral intentions. This indicates the importance of the awareness of consequence, ascribed responsibility, anticipated pride, and anticipated guilt constructs in determining people’s personal norm and, subsequently, their pro-mandatory vaccination-friendly behavior.

Mass media coverage of the COVID-19 vaccine was found to have a positive and significant effect on the awareness of consequence and intention to take the COVID-19 vaccine before international traveling. This is most likely due to the mass media narrative of the COVID-19 vaccination program, which showed robust capabilities in delivering valuable information, suppressing misinformation, and to a certain level, improving public knowledge on the COVID-19 vaccine program. Furthermore, the individual’s information processing related to the COVID-19 vaccine program was driven by one’s feelings of fear and hope. Hence, mass media messages that were built on hope and enthusiasm towards the COVID-19 vaccine have positively affected people’s intention to take the COVID-19 vaccine before traveling internationally. As such, mass media messages engaged people in asking questions and learning more about vaccination programs. On the other hand, mass media messages that were built on anxiety and depression have positively affected awareness of consequences, as people engaged to act responsibly out of fear for their own and other people’s health.

### 5.1. Theoretical Implications

The novelty of the present study is that it is the first study to assess the behavioral intentions of travelers towards taking mandatory COVID-19 vaccines as a precondition for international travel. Two main theoretical implications can be concluded from the findings of this study. First, our conceptual model has successfully extended the NAM and has displayed a robust explanatory power in understanding the behavioral intentions of travelers towards taking a mandatory COVID-19 vaccine as a precondition for international travel. From a tourist behavior stance, the present study has offered a novel vantage point into what Perugini and Bagozzi [53] referred to as building up and widening the modern social theory in tourist-behavior literature. More precisely, the study results offer valuable insights into the behavioral intentions of international travelers by extending the existing NAM framework through the mass media coverage of the COVID-19 vaccine.

Second, the current study has demonstrated the significant role of personal norm as a mediator in travelers’ behavioral intentions towards taking a mandatory COVID-19 vaccine as a precondition for international travel. Specifically, the results of the SEM are strongly consistent with the NAM model. Personal norm had a robust relationship with the behavioral intentions of travelers towards taking a mandatory COVID-19 vaccine as a precondition for international travel (β 0.943, *p* < 0.001). Furthermore, the mass media coverage demonstrated a positive influence on travelers’ behavioral intentions towards taking a mandatory COVID-19 vaccine as a precondition for international travel (β 0.063, *p* < 0.001). Overall, the set of predictors from the extended NAM model together accounted for 89% of the variance of behavioral intention. Accordingly, from a theoretical standpoint, the authors have successfully extended the original NAM in line with the benchmark for enhancing the theory. However, this study’s results revealed that ascribed responsibility did not positively influence international travelers’ anticipated pride. Accordingly, in the cases where individuals are stripped from freedom of choice and sovereignty over themselves, the feeling of pride from ascribed responsibility disappears.

### 5.2. Practical Implications

In modern liberal society, freedom of choice is a principal value. Hence, government-enforced social control (COVID-19 mandatory vaccination as a precondition to international travel) cannot violate individuals’ freedom without certain consequences. Accordingly, a COVID-19 mandatory vaccination as a precondition to international travel has to emerge and be maintained in such a way that people still consider themselves to have freedom of choice.

The awareness of consequences related to the COVID-19 and its implications on restricted international traveling seems to elicit ascribed responsibility. Accordingly, governments should engage in well-designed campaigns to educate their citizens in regards to the benefits of COVID-19 vaccines so the potentially misleading anti-vaccine sentiments do not prevail. This can be achieved by prompting international travelers to accept the mandatory COVID-19 vaccination through transparency and government support. Furthermore, as the ascribed responsibility affects the feeling of guilt and personal norm to take the COVID-19 vaccine, governments should also explore possibilities on how to elicit the feeling of obligation to take COVID-19 vaccine. Consequently, feeling of obligation (personal norms) towards close family members, significant other, elderly people, immunocompromised and fellow humans in general would create a sympathy and passion towards altruistic behavior. Thus, international travelers would diminish their own self-interest and cooperate for the benefits of public health. Lastly, although the international travelers’ intention to take the COVID-19 vaccine is influenced by personal norms and mass media coverage, it appears that such intention is rooted in international travelers’ fear of social sanctions and social exclusions. Accordingly, governments in liaison with the pharmaceutical industry should provide certain guarantees for their COVID-19 vaccines; fully transparent information about the real effectiveness of the vaccine (based on precisely defined parameters of the immune response), and take ethical and financial responsibility in case of adverse reaction to their COVID-19 vaccines. On the other hand, governments have to engage with people in an open dialogue (via mass media) to create an atmosphere of mutual trust. Consequently, mutual trust would enhance individuals’ moral responsibility without creating the conflicting feeling of lost freedom of choice. In this way, international travelers would with no difficulty accept the mandatory COVID-19 vaccination as their sympathy for the social group/groups and for the good of society as a whole would enhance their pro-socially behavior.

### 5.3. Limitations and Future Research

The present study is not without certain limitations that can offer opportunities for future research. The first limitation is the use of an online self-administered survey. Hence, a careful watch should be taken in generalizing the findings due to self-response bias. Nevertheless, in the present study, the authors have designed a survey following the suggestion outlined by Podsakoff et al. [42]. Thus, the issue with self-response bias was lessened in such a way that independent and dependent variables did not replicate the structure of the hypotheses. Also, future studies should employ a field survey method to overcome the aforementioned limitation once actual widespread COVID-19 vaccination begins. The second limitation is the study design, which was cross-sectional. Consequently, as Carlson and Morrison [54] argued, there is no time-related correlation between exposure and outcome. Future studies should employ a longitudinal study design to overcome the limitation of the present study. Lastly, this study involved participants who were international tourists with skills and access to use the internet. Accordingly, future studies should include international tourists who do not have skills to use the internet or who do not have access to the internet. Notwithstanding these limitations, to the authors’ best knowledge, the present study is the first to investigate travelers’ behavioral intentions to take a mandatory COVID-19 vaccine before international travel.

## Figures and Tables

**Table 1:** List of social media groups from where participants were recruited.

Social Media Group	Domain
China Travel Group	https://www.facebook.com/groups/164125967451164
Tourists	https://www.facebook.com/groups/642485595801073
Tripadvisor Travel Forum	https://www.tripadvisor.com/ForumHome
Thorn Tree forum	https://www.lonelyplanet.com/thorntree/welcome
Fodor’s Travel Talk Forums	https://www.fodors.com/community/trending.php
Travel and Tourism	https://www.facebook.com/groups/433024676868583
Worldwide Travel	https://www.facebook.com/groups/worldwidetravel
Travellers Around The World	https://www.facebook.com/groups/885989791516753
Travellers point	https://www.travellerspoint.com/forum.cfm
South Asian Tourism & Travelers Group	https://www.facebook.com/groups/1504362079863590

**Table 2:** Demographic characteristic of sample (*n* = 1221).

Variable	*n*	%
Gender		
Male	590	48.3
Female	631	51.7
Age group		
20–29	485	39.7
30–40	374	30.6
41–50	277	22.7
51–60	61	5.0
60 and older	24	2.0
Education level		
High school	274	22.4
Associate degree	252	20.6
Bachelor’s degree	492	40.3
Master’s or doctoral degree	203	16.7
Place of residence		
North America	137	11.2
Central/South America	122	10.0
Europe	125	10.2
China	215	17.6
South Asia	170	13.9
South East Asia	232	19.0
Africa	209	17.1
Australia and New Zealand	11	1.0

**Table 3:** Measurement model assessment (*n* = 1221).

Variable	Mean	SD	Skewness	Kurtosis	Standardized Factor Loading	Composite Reliability	Cronbach’s α
MAC						0.857	0.847
MAC1	3.24	1.239	−0.374	−0.856	0.771		
MAC2	3.24	1.177	−0.325	−0.726	0.954		
AWC						0.848	0.846
AWC1	3.74	1.034	−0.651	0.008	0.813		
AWC2	3.92	0.909	−0.751	0.524	0.808		
AWC3	3.8	0.956	−0.615	0.048	0.797		
ACR						0.898	0.898
ACR1	3.73	1.002	−0.624	0.069	0.875		
ACR2	3.73	0.994	−0.635	0.109	0.845		
ACR3	3.8	1.021	−0.655	−0.011	0.87		
PSN						0.916	0.916
PSN1	3.18	1.275	−0.23	−0.98	0.914		
PSN2	3.19	1.277	−0.248	−0.965	0.924		
ANP						0.945	0.945
ANP1	3.14	1.29	−0.174	−1.015	0.918		
ANP2	3.06	1.273	−0.125	−1.013	0.927		
ANP3	3.17	1.297	−0.225	−1.038	0.925		
ANG						0.925	0.925
ANG1	2.33	1.09	0.515	−0.421	0.882		
ANG2	2.31	1.07	0.5	−0.409	0.919		
ANG3	2.33	1.109	0.548	−0.425	0.89		
BHI						0.941	0.94
BHI1	3.17	1.268	−0.224	−1.002	0.919		
BHI2	3.17	1.271	−0.224	−0.986	0.905		
BHI3	3.13	1.267	−0.162	−0.986	0.927		

Note 1. All factor loadings statistically significant at *p* < 0.001; Note 2. MAC = Mass media coverage; AWC = Awareness of consequences; ACR = Ascribed responsibility; PSN = Personal norm; ANP = Anticipated pride; ANG = Anticipated guilt; BHI = Behavioral intention.

**Table 4:** The results of the CFA model and factor correlations (*n* = 1221).

Variable	1	2	3	4	5	6	7	AVE	(√AVE)
1. MAC	1.000							0.752	(0.867)
2. AWC	0.185	1.000						0.650	(0.806)
3. ACR	0.171	0.821	1.000					0.746	(0.864)
4. PSN	0.326	−0.024	0.027	1.000				0.845	(0.919)
5. ANP	0.345	−0.019	0.004	0.936	1.000			0.853	(0.924)
6. ANG	−0.087	−0.506	−0.411	0.193	0.136	1.000		0.805	(0.897)
7. BHI	0.345	−0.044	−0.043	0.942	0.897	0.179	1.000	0.841	(0.917)
*M*	3.24	3.82	3.75	3.37	3.12	2.32	3.16		
*SD*	1.125	0.846	0.916	0.948	1.222	1.016	1.199		

Note 1. Goodness-of-fit indices: χ^2^ (131) = 625.377, *p* < 0.001, RMSEA = 0.056 [0.051, 0.060], CFI = 0.977, TLI = 0.970, SRMR = 0.019. Note 2. MAC = Mass media coverage; AWC = Awareness of consequences; ACR = Ascribed responsibility; PSN = Personal norm; ANP = Anticipated pride; ANG = Anticipated guilt; BHI = Behavioral intention; CFA = confirmatory factor analysis.

**Table 5:** The results of fully latent structural regression model (*n* = 1221).

Relationships								Estimate	S.E.	Est./S.E.
H1:	AWC		→		ACR			0.869 (0.833)	0.032	27.344 ***
H2:	ACR		→		PSN			0.062 (0.047)	0.022	2.760 **
H3:	PSN		→		BHI			0.929 (0.943)	0.020	45.412 ***
H4:	ACR		→		ANP			−0.006 (−0.005)	0.042	−0.153
H5:	ANP		→		PSN			0.913 (0.938)	0.020	46.087 ***
H6:	ACR		→		ANG			−0.481 (−0.439)	0.033	−14.546 ***
H7:	ANG		→		PSN			0.107 (0.089)	0.020	5.459 ***
H8:	MAC		→		AWC			0.164 (0.194)	0.028	5.819 ***
H9:	MAC		→		BHI			0.072 (0.063)	0.018	3.976 ***
Direct effect:	AWC			→			BHI	−0.064 (−0.047)	0.021	−2.990 **
Indirect effect:	AWC	→	ACR	→	PSN	→	BHI	0.050 (0.037)	0.018	2.754 **

Note 1. * *p* < 0.05 (This data means that test was done even though there is no result), ** *p* < 0.01, *** *p* < 0.001. Note 2. Standardized values are in parentheses; Bias-corrected bootstrap sample size = 5000. Note 3. Goodness-of-fit indices: χ^2^ (141) = 888.142, *p* < 0.001, RMSEA = 0.066 [0.062, 0.070], CFI = 0.965, TLI = 0.958, SRMR = 0.084. Note 4. MAC = Mass media coverage; AWC = Awareness of consequences; ACR = Ascribed responsibility; PSN = Personal norm; ANP = Anticipated pride; ANG = Anticipated guilt; BHI = Behavioral intention; Note 5. Total variance explained (R^2^): AWC = 0.038; ACR = 0.694; PSN = 0.887; ANP = 0.000; ANG = 0.193; BHI = 0.893.

## Data Availability

The dataset used in this research are available upon request from the corresponding author. The data are not publicly available due to restrictions i.e., privacy or ethical.

## Appendix A

**Table A1:** Constructs and Measurement Items.

Constructs and Items
**Mass media coverage** Media coverage (TV, newspapers, online) convey a positive image of COVID-19 vaccine. Media coverage makes me want to take COVID-19 vaccine.
**Awareness of consequences** The COVID-19 outbreak and pandemic is more serious than what individuals think in the tourism industry. I concern that COVID-19 and its impact on the tourism industry lasts longer than we expect. I am aware of the seriousness of COVID-19 and its considerable influence on the tourism industry.
**Ascribed responsibility** I believe that every traveler is partly responsible for the COVID-19 outbreak and pandemic. I feel that every traveler is jointly responsible for the COVID-19 outbreak and pandemic. Every traveler must assume responsibility for the COVID-19 outbreak and pandemic.
**Personal norm** I feel morally obliged to take COVID-19 vaccine as precondition of international traveling when available. I feel personally obliged to take COVID-19 vaccine as precondition of international traveling when available. I feel a moral obligation to take COVID-19 vaccine as precondition of international traveling when available.
**Anticipated pride** *Imagine that, you take COVID-19 vaccine as precondition of international traveling when available. How would you feel?* I would feel proud. I would feel accomplished. I would feel confident.
**Anticipated guilt** *Imagine that, you take COVID-19 vaccine as precondition of international traveling when available. How would you feel?* I would feel guilty. I would feel remorseful. I would feel sorry.
**Behavioral intention** I am willing to take COVID-19 vaccine as precondition of international traveling when available. I plan to take COVID-19 vaccine as precondition of international traveling when available. I will spend my effort on taking COVID-19 vaccine as precondition of international traveling when available.
